# Supplementary material for: Dopamine D2 receptor agonists abrogate neuroendocrine tumour angiogenesis to inhibit chemotherapy-refractory small cell lung cancer progression
Source: Cell Death Dis. 2025 May 9;16(1):370. doi: 10.1038/s41419-025-07693-y (PMC12064713; doi:10.1038/s41419-025-07693-y)
Supplement: Supplementary file 1 — Supplementary Figures 1–9 and Supplementary Table 1 [file 41419_2025_7693_MOESM1_ESM.pdf]

Dopamine D_2 receptor agonists abrogate neuroendocrine tumour angiogenesis to inhibit chemotherapy-refractory small cell lung cancer progression

Supplementary Figures 1-9 and Supplementary Table 1:

Supplementary Figure 1: Pre- and post-treatment luminescence images of vehicle- and quinpirole-treated SCID mice.

Supplementary Figure 2: Full, uncropped images of immunoblots corresponding to Figure 2i-j.

Supplementary Figure 3: D_2R agonist Cabergoline inhibits tube formation through apoptosis of human endothelial cells, and this effect can be reversed through inhibition of apoptosis.

Supplementary Figure 4: D_2R agonist Cabergoline reduces endothelial cell proliferation.

Supplementary Figure 5: D_2R agonist Cabergoline reduces human endothelial cell migration.

Supplementary Figure 6: Validation of D_2R knockdown in endothelial cells.

Supplementary Figure 7: Conditioned medium from human endothelial cells treated with a D_2R agonist increases caspase-3–mediated apoptosis of SCLC chemotherapy-resistant organoids.

Supplementary Figure 8: Treating human SCLC PDX organoids with D_2R agonist quinpirole may contribute to an enhanced $CD8^+$ T cell response in the SCLC immune microenvironment.

Supplementary Figure 9: Co-immunofluorescence images of D_2R and CD31 immunostaining in chemonaïve and chemoresistant SCLC PDXs.

Supplementary Table 1: D_2R protein expression in tumour-associated endothelial cells in paired chemotherapy-naïve and chemotherapy-resistant patient specimens.

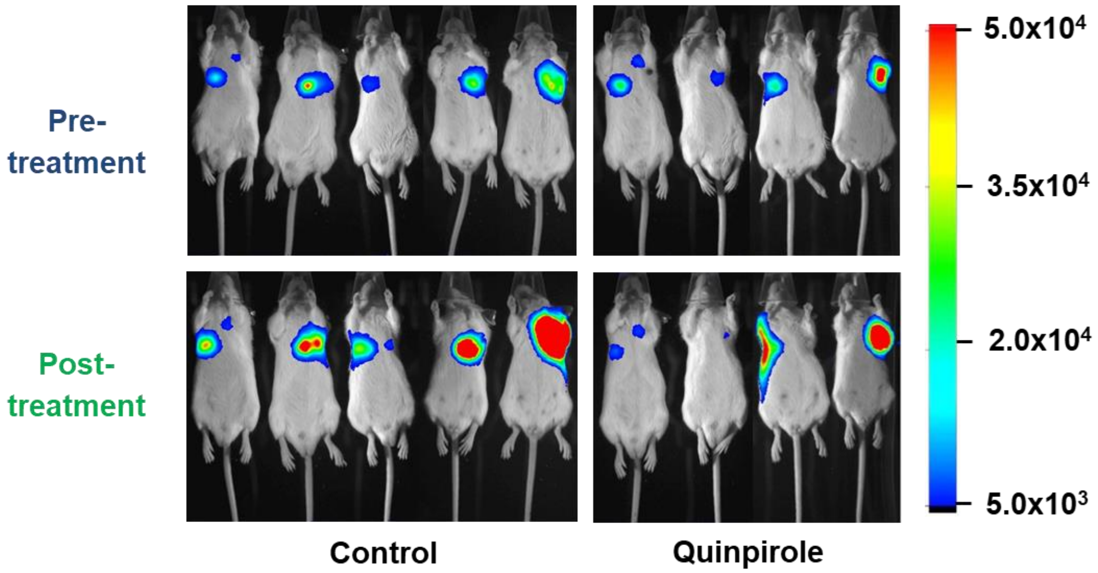

Supplementary Figure 1: Pre- and post-treatment luminescence images of vehicle- and quinpirole-treated SCID mice. One million luciferase-labelled human DMS-53 SCLC cells were orthotopically injected into the left thoracic cavity of SCID mice. Mice administered either vehicle control (1× PBS) or quinpirole (10 mg/kg) were imaged for bioluminescence before and after the treatment.

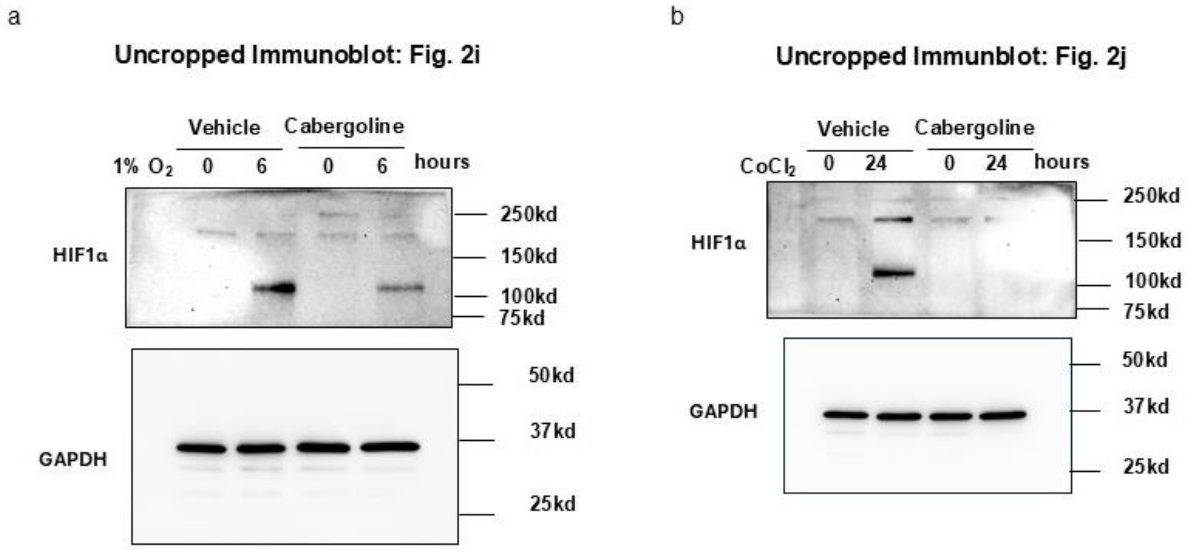

Supplementary Figure 2: Full, uncropped images of immunoblots corresponding to Figure 2i-j. a. The full, uncropped immunoblots corresponding to Figure 2i are depicted. **b.** The full, uncropped immunoblots associated with Figure 2j are shown.

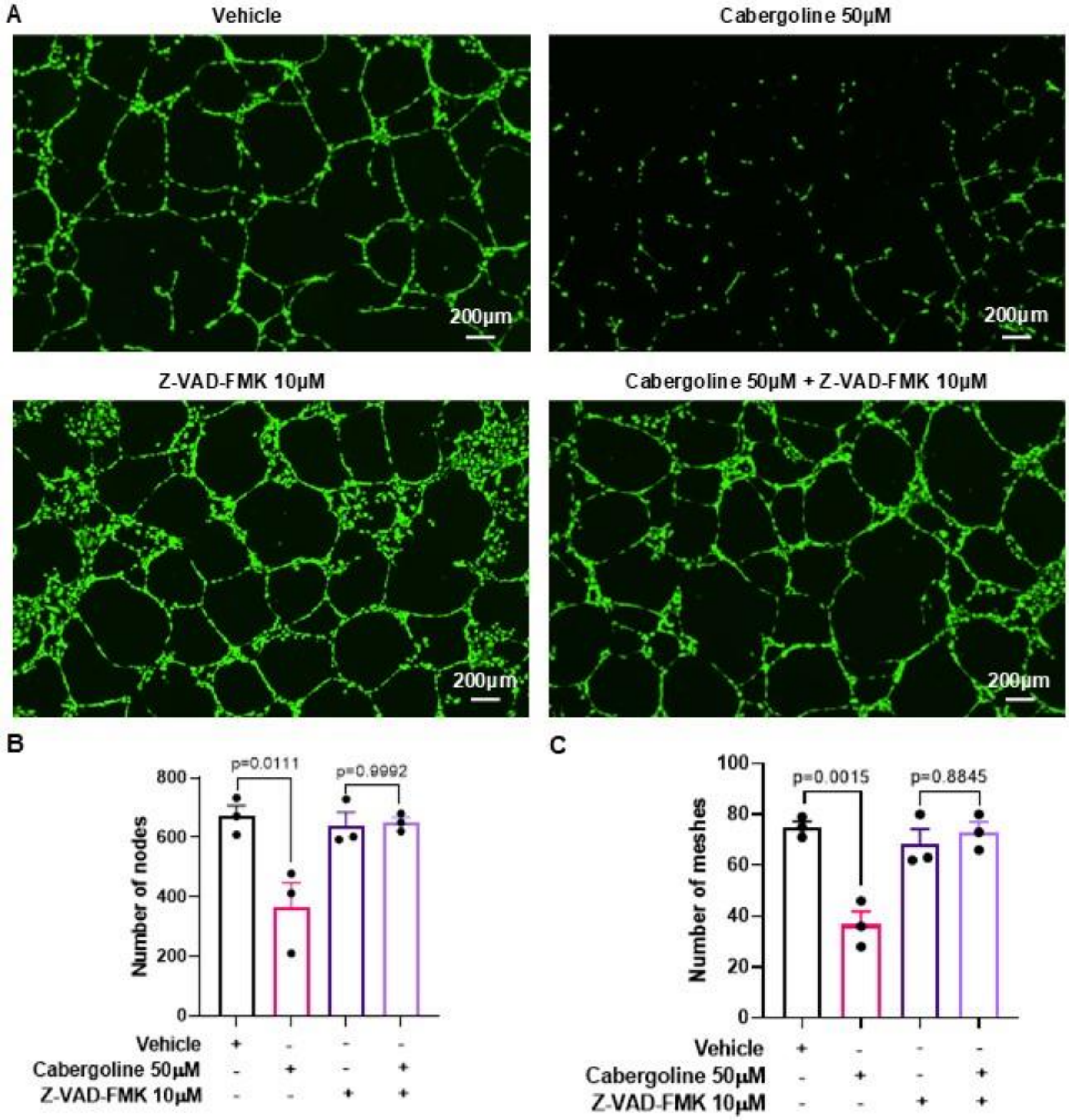

Supplementary Figure 3: D_2R agonist Cabergoline inhibits tube formation through apoptosis of human endothelial cells, and this effect can be reversed through inhibition of apoptosis. a. HUVEC were cultured in 24-well plates coated with Matrigel and treated with DMSO vehicle, 50 μM Cabergoline, 10 μM apoptosis inhibitor Z-VAD-FMK, or both 50 μM Cabergoline and 10 μM apoptosis inhibitor Z-VAD-FMK. At 16 h post-seeding, 8 μg/ml Calcein AM was added, and imaging was performed 30 min later using an Incucyte with a 4× objective. **b-c.** The number of nodes (b) and number of meshes (c) were affected by D2R agonist, which were rescued by apoptosis inhibitor. One-way ANOVA analysis followed by Tukey's multiple comparisons test was performed.

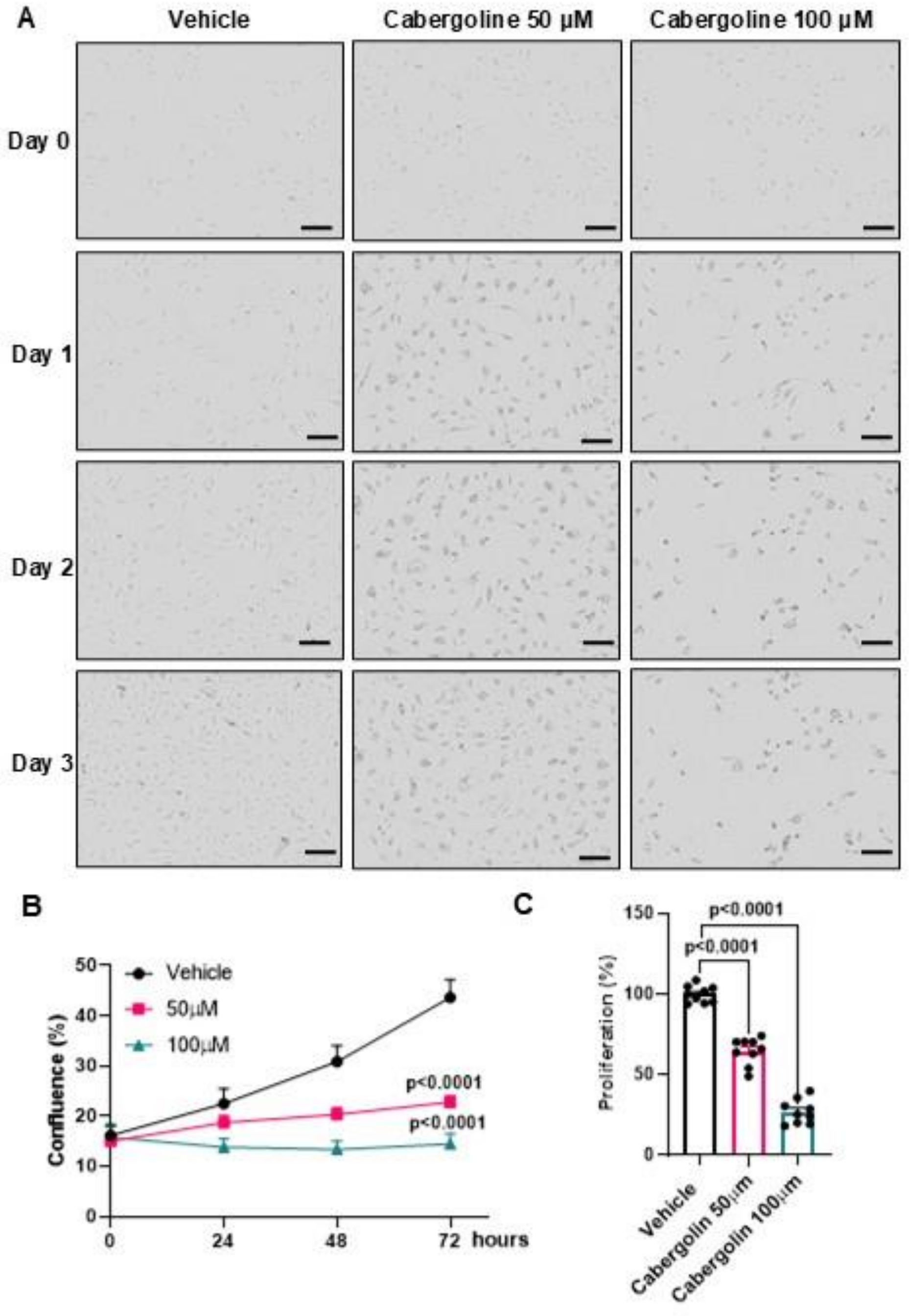

Supplementary Figure 4: D_2R agonist Cabergoline reduces endothelial cell proliferation. a. HUVEC were seeded at a concentration of 1,000 cells per well in a 96-well plate. Cells were treated with vehicle or Cabergoline at the indicated concentration after 24 hours and transferred to an Incucyte S3 to monitor the growth of cells by capturing images with a 10x objective every 8 hours. Scale bar: 200 µM. **b.** The confluence of cells was analyzed using immortalized AI confluence segmentation. Two-way ANOVA analysis followed by Dunnett's multiple comparisons test was performed to separate the differences. **c.** At the end of the experiment, cell proliferation was quantified using CellTiter-Glo® 2.0 Cell Viability Assay by measuring the amount of ATP present. 3 independent biological repeats and 3 technical repeats were reported. One-way ANOVA analysis followed by Dunnett's multiple comparisons test was performed.

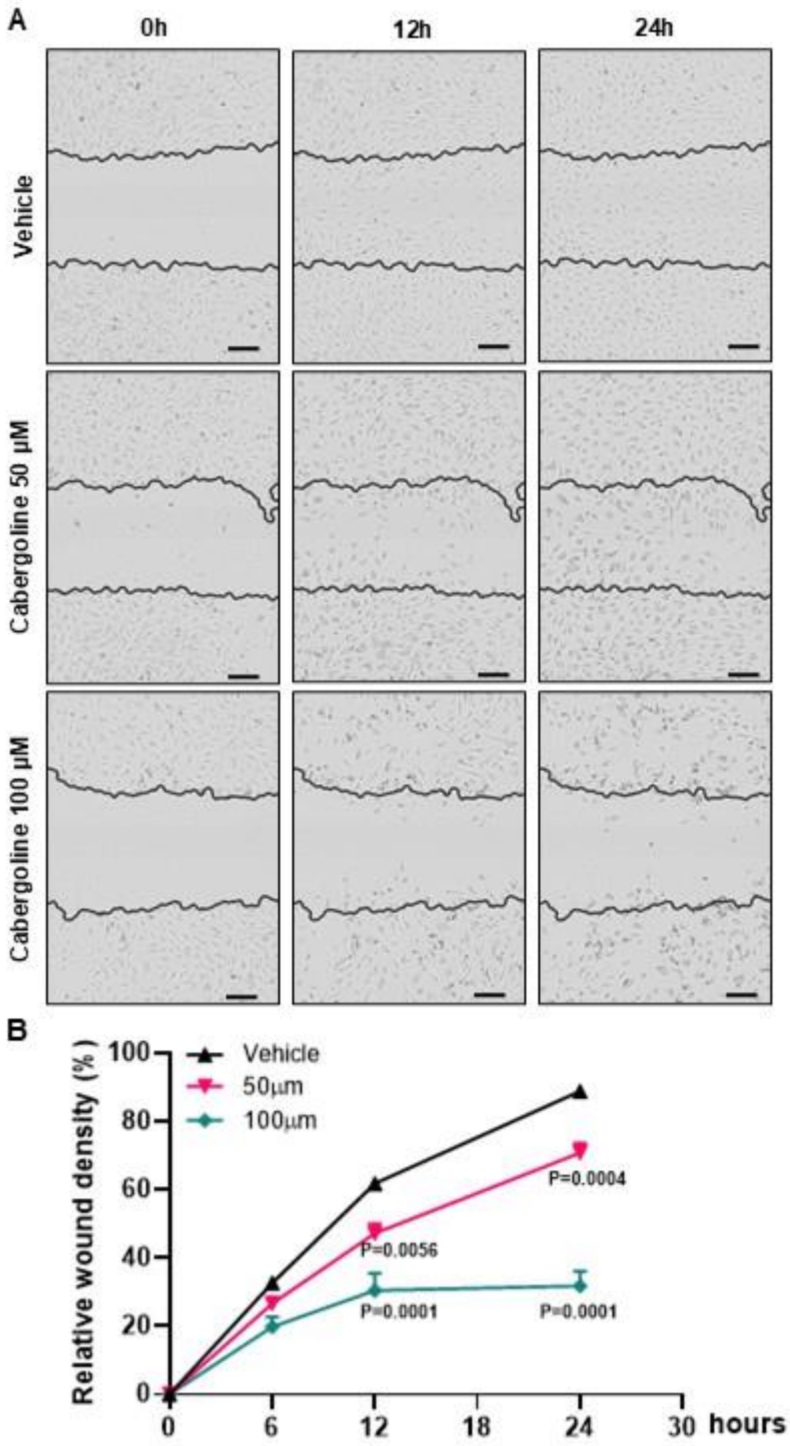

Supplementary Figure 5: D_2R agonist Cabergoline reduces human endothelial cell migration. A wound-healing assay was performed in HUVEC by creating a scratch with the Incucyte 96-well wound-making tool, and images were captured after 6 h, 12 h and 24 h using an Incucyte S3 with a 4× objective. 50 µM and 100 µM Cabergoline reduced relative wound density at 12 h and 24 h. Scale: 200 µm. Two-way ANOVA analysis followed by Bonferroni's multiple comparisons test was performed to separate the differences.

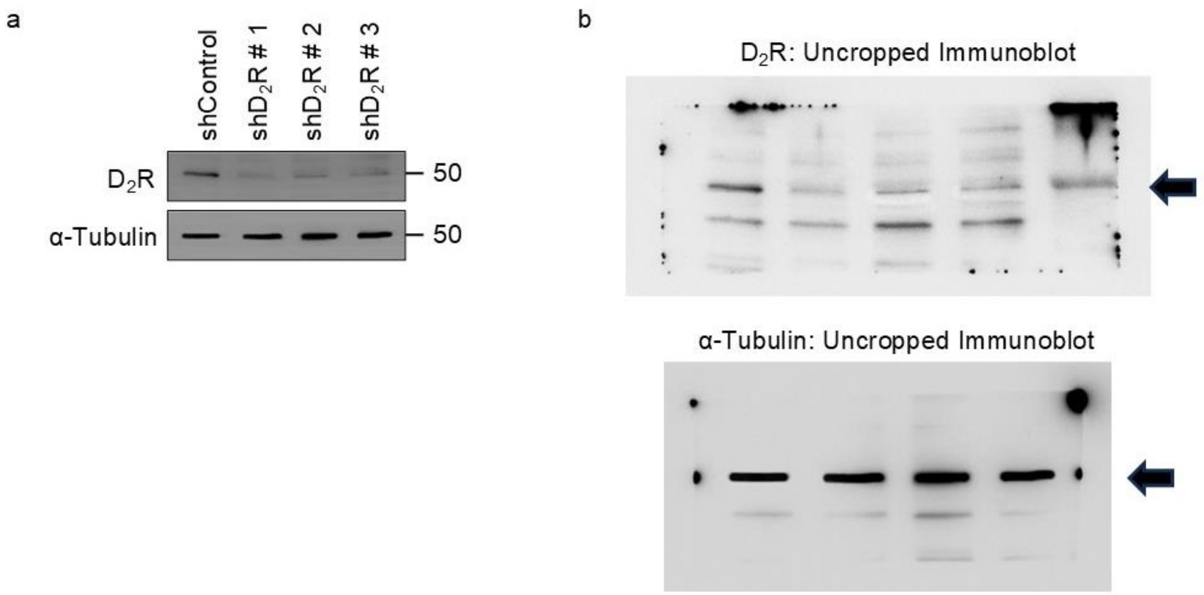

Supplementary Figure 6: Validation of D_2R knockdown in endothelial cells. a. HUVEC transduced with lentivirus encoding either a D_2R shRNA (#1-3) or control shRNA were lysed. Equal amounts of protein were separated in a 4-20% SDS-PAGE gel followed by protein transfer to PVDF membrane. Antibody-reactive bands were detected using primary antibodies against D_2R and α-tubulin (loading control). **b.** The full, uncropped immunoblots are depicted. The arrows indicate the bands corresponding to the proteins of interest.

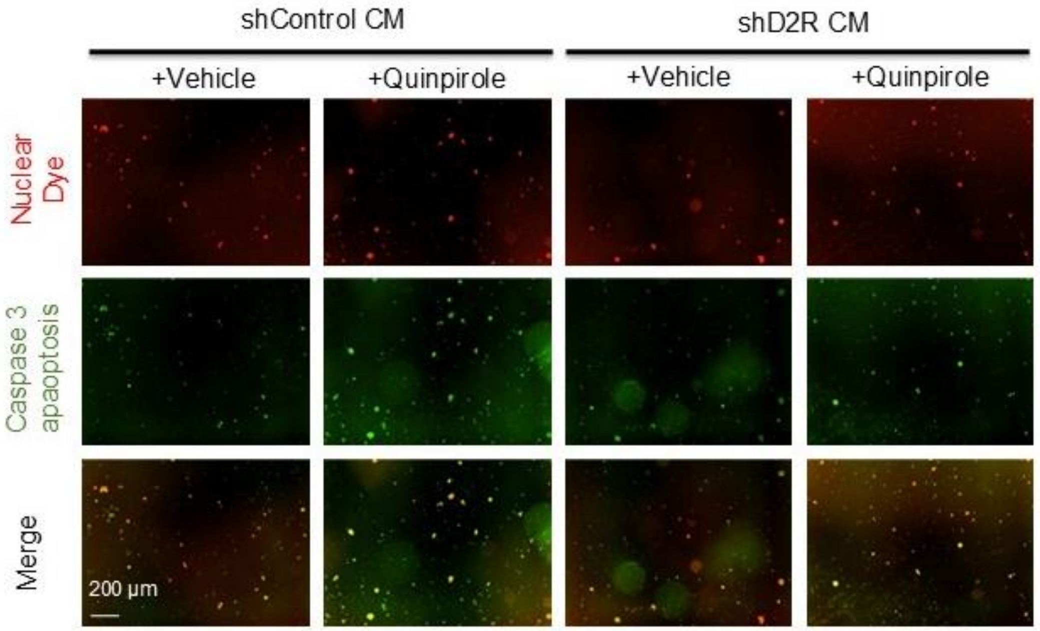

Supplementary Figure 7: Conditioned medium from human endothelial cells treated with a D_2R agonist increases caspase-3–mediated apoptosis of SCLC chemotherapy-resistant organoids. SCLC PDX cultured as three-dimensional organoids showed increased apoptosis upon treatment with conditioned media collected from quinpirole (50 µM)-treated HUVEC.

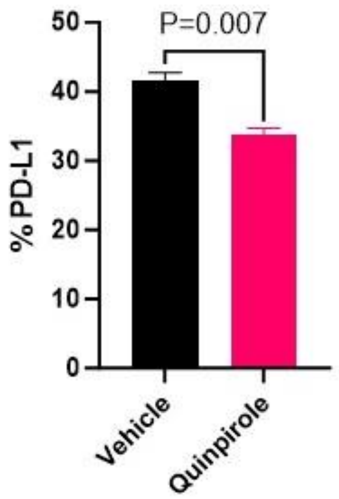

Supplementary Figure 8: Treating human SCLC PDX organoids with D_2R agonist quinpirole may contribute to an enhanced $CD8^+$ T cell response in the SCLC immune microenvironment. JHU-LX33R SCLC PDX organoids were treated with vehicle or D_2R agonist quinpirole (50 μM) for 72 hours and subjected to flow cytometry using fluorophore conjugated antibodies to detect PD-L1. Two independent biological repeats and 4 technical repeats were performed. One-way ANOVA followed by Tukey's multiple comparisons test was performed.

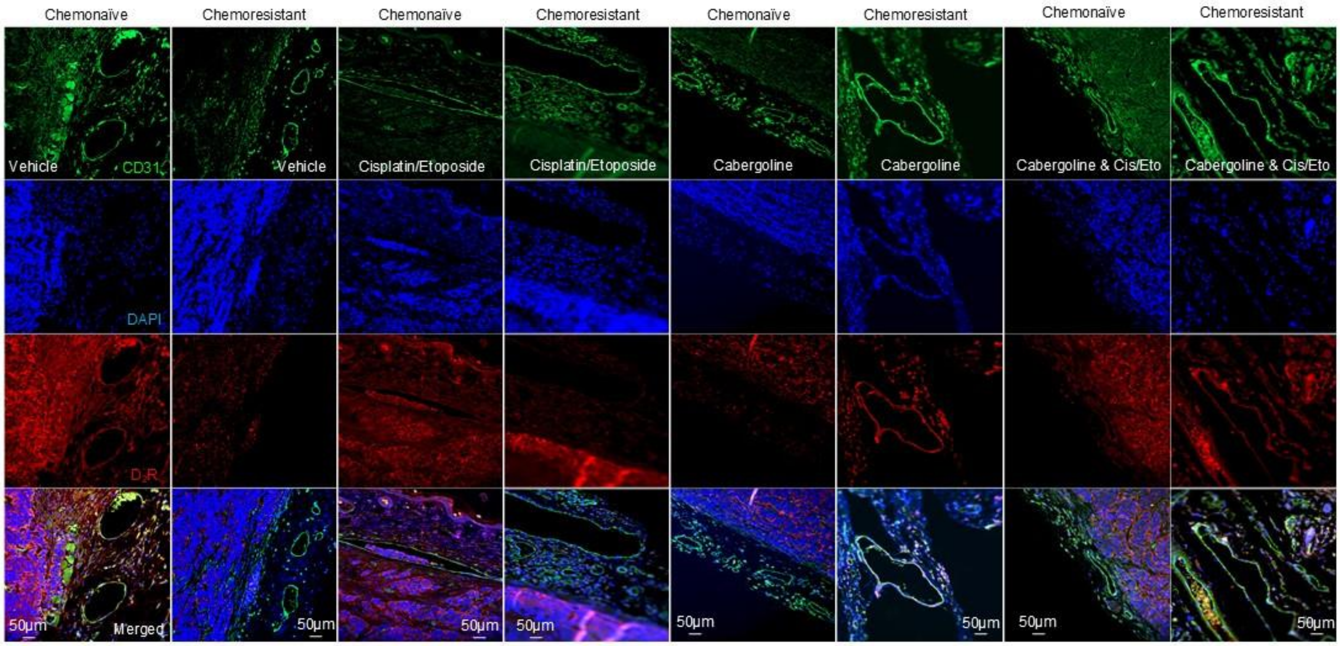

Supplementary Figure 9: Co-immunofluorescence images of D_2R and CD31 immunostaining in chemonaïve and chemoresistant SCLC PDXs. NSG mice were subcutaneously implanted with 5×10^6 cells obtained from either chemonaïve (MSK-LX40) or chemoresistant (MSK-LX40R) human SCLC PDXs. Mice were randomly divided into four groups to receive 1) vehicle; 2) cisplatin/etoposide; 3) cabergoline; or 4) cabergoline and cisplatin/etoposide. At the endpoint, mice were sacrificed, tumours were resected, and co-immunofluorescence staining was performed on FFPE tumour tissues (n=3 per group) using primary antibodies against CD31 and D_2R. Nuclei were counterstained with DAPI.

Supplementary Table 1: D_2R protein expression in tumour-associated endothelial cells in paired chemotherapy-naïve and chemotherapy-resistant patient specimens

Patient Number	Subtype	Chemotherapy Status	D_2R Staining in Endothelium
4	SCLC-A	Chemotherapy-naïve	40%
4	SCLC-A	Chemotherapy-resistant	1%
7	SCLC-A	Chemotherapy-naïve	1%
7	SCLC-A	Chemotherapy-resistant	30%
16	SCLC-A	Chemotherapy-naïve	20%
16	SCLC-A	Chemotherapy-resistant	5%
17	SCLC-A	Chemotherapy-naïve	50%
17	SCLC-A	Chemotherapy-resistant	30%
22	SCLC-A	Chemotherapy-naïve	40%
22	SCLC-A	Chemotherapy-resistant	60%
25	SCLC-A	Chemotherapy-naïve	30%
25	SCLC-A	Chemotherapy-resistant	1%
29	SCLC-A	Chemotherapy-naïve	50%
29	SCLC-A	Chemotherapy-resistant	5%
32	SCLC-A	Chemotherapy-naïve	20%
32	SCLC-A	Chemotherapy-resistant	10%
34	SCLC-A	Chemotherapy-naïve	50%
34	SCLC-A	Chemotherapy-resistant	1%
