# Supplementary material for: Dopamine D2 receptor agonists abrogate neuroendocrine tumour angiogenesis to inhibit chemotherapy-refractory small cell lung cancer progression
Source: Cell Death Dis. 2025 May 9;16(1):370. doi: 10.1038/s41419-025-07693-y (PMC12064713; doi:10.1038/s41419-025-07693-y)
Supplement: Supplementary file 2 — Original Data - Uncropped Immunoblots [file 41419_2025_7693_MOESM2_ESM.pdf]

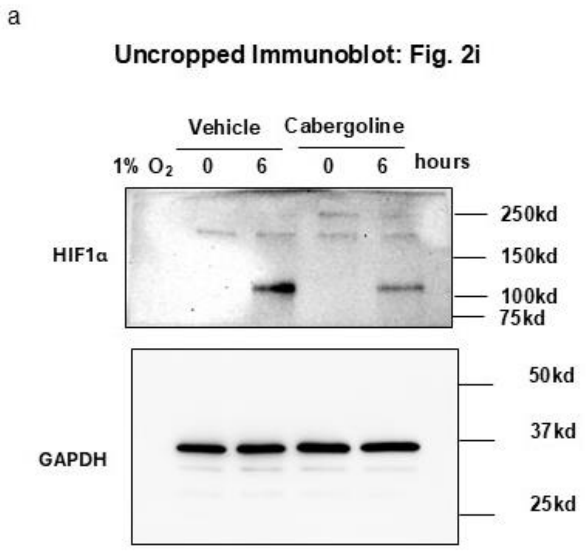

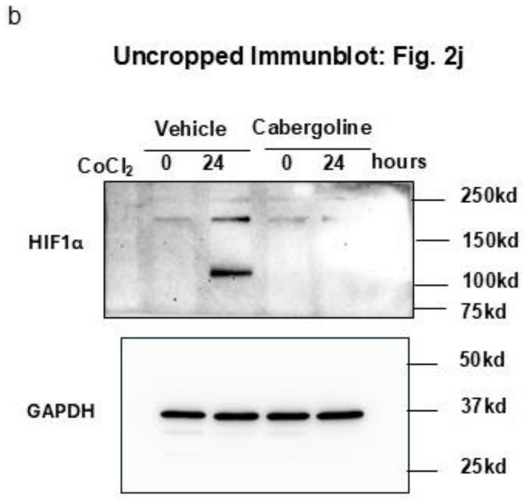

Supplementary Figure 2: Full, uncropped images of immunoblots corresponding to Figure 2i-j. a. The full, uncropped immunoblots corresponding to Figure 2i are depicted. **b.** The full, uncropped immunoblots associated with Figure 2j are shown.

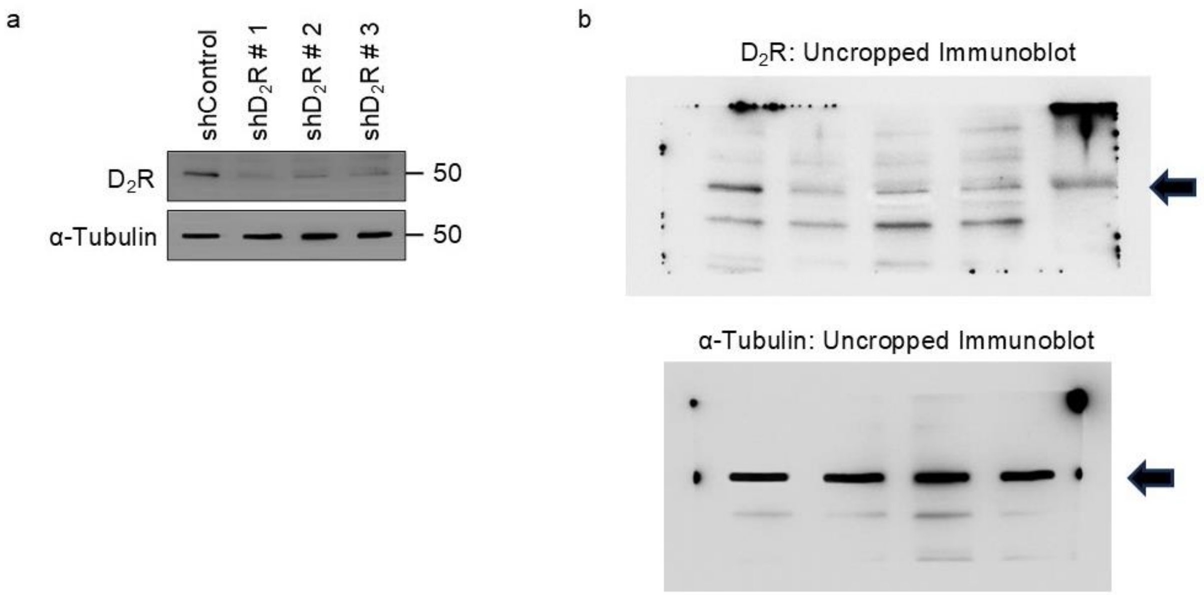

Supplementary Figure 6: Validation of D_2R knockdown in endothelial cells. a. HUVEC transduced with lentivirus encoding either a D_2R shRNA (#1-3) or control shRNA were lysed. Equal amounts of protein were separated in a 4-20% SDS-PAGE gel followed by protein transfer to PVDF membrane. Antibody-reactive bands were detected using primary antibodies against D_2R and α-tubulin (loading control). **b.** The full, uncropped immunoblots are depicted. The arrows indicate the bands corresponding to the proteins of interest.
